# Association between violence and anxiety and depression problems in women living in the Magdalena region, Colombia

**DOI:** 10.1192/j.eurpsy.2023.1356

**Published:** 2023-07-19

**Authors:** K. Múnera-Luque, U. Rodríguez-De Ávila

**Affiliations:** Cognition and Education Research Group, Universidad del Magdalena, Santa Marta, Colombia

## Abstract

**Introduction:**

Violence against women constitutes a social and health problem, exploring the impact that this can generate on mental health is an indispensable resource for the development of intervention and prevention strategies, primarily in one of the regions with the highest report of femicides in Colombia, such as Magdalena.

**Objectives:**

To evaluate the association between violence and anxiety and depression problems in women living in the Magdalena region, Colombia.

**Methods:**

The study was quantitative, exploratory and by convenience, with face-to-face application and by web platform. The sample consisted of 375 women residents (x̄=32; sd=13) in the Magdalena region -Colombia. Psychometric tests adapted and validated in Colombia and Mexico were applied, with Cronbach’s alpha values between .81 and .95, to evaluate anxiety (Self-Rating Anxiety Scale by Zung, 1971, adapted by Rodríguez et al, 2020), depression (Abbreviated Scale of the Center for Epidemiological Studies of Depression-10. by Radloff, 1977, abbreviated and validated by Rueda-Jaimes et al., 2009), and violence (Suffered and Exercised Partner Violence Questionnaire by De la Rubia and Sandra, 2015). In the data analysis, a nonparametric distribution was identified. Spearman was used to estimate correlations and the Kruskal-Wallis test was used to verify intergroup variance.

**Results:**

Of the total sample, 40.3% showed medium depression, 67.5% showed low anxiety, 3.2% showed a high degree of suffered violence and 2.9% showed a high degree of exercised violence.Significant positive correlations were found between violence, anxiety and depression, as well as between violence, anxiety and depression (see Figure 1). Significant differences were also found between the variables (see Figure 2).

**Image:**

**Figure:**
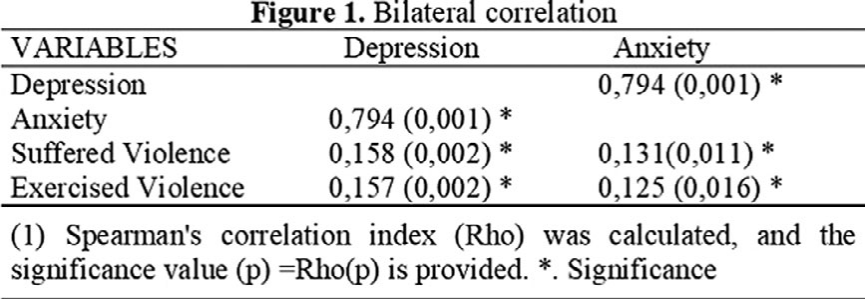

**Image 2:**

**Figure:**
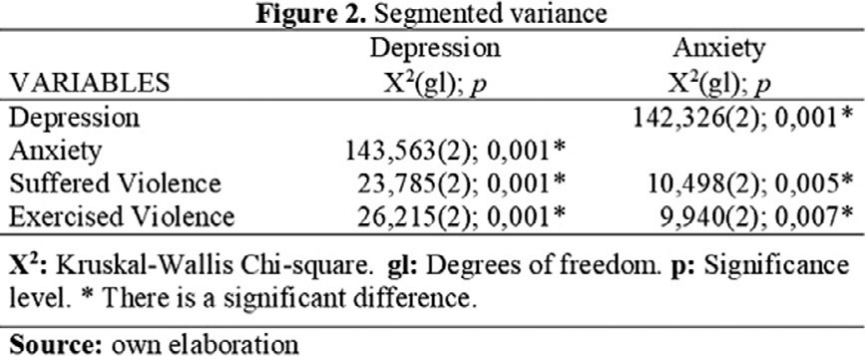

**Conclusions:**

The results allow us to conclude that violence against women may be associated with anxiety and depression problems in this population, which allows us to explore this phenomenon from a public health perspective. This also allows us to contemplate the importance of devoting greater efforts to its prevention. Credit is given to project BPIN 2020000100758: Development of an Integrated Technological System for the promotion of mental health, psychosocial and socioemotional problems and prevention of gender violence caused by the COVID19 pandemic in the Magdalena region, and to Universidad del Magdalena for their support and funding.

**Disclosure of Interest:**

None Declared

